# Current State of Evidence: Influence of Nutritional and Nutrigenetic Factors on Immunity in the COVID-19 Pandemic Framework

**DOI:** 10.3390/nu12092738

**Published:** 2020-09-08

**Authors:** Sebastià Galmés, Francisca Serra, Andreu Palou

**Affiliations:** 1 Laboratory of Molecular Biology, Nutrition and Biotechnology, NUO Group, Universitat de les Illes Balears, 07122 Palma, Spain; s.galmes@uib.cat (S.G.); andreu.palou@uib.es (A.P.); 2 CIBER de Fisiopatología de la Obesidad y Nutrición (CIBEROBN), 28029 Madrid, Spain; 3 Institut d’Investigació Sanitària Illes Balears (IdISBa), 07120 Palma, Spain; 4 Alimentómica S.L., Spin-off n.1 of the University of the Balearic Islands, 07121 Palma, Spain

**Keywords:** immunity, micronutrient, COVID-19, SARS-CoV-2, genetic variant, nutrigenetics, epidemiology

## Abstract

The pandemic caused by the new coronavirus has caused shock waves in many countries, producing a global health crisis worldwide. Lack of knowledge of the biological mechanisms of viruses, plus the absence of effective treatments against the disease (COVID-19) and/or vaccines have pulled factors that can compromise the proper functioning of the immune system to fight against infectious diseases into the spotlight. The optimal status of specific nutrients is considered crucial to keeping immune components within their normal activity, helping to avoid and overcome infections. Specifically, the European Food Safety Authority (EFSA) evaluated and deems six vitamins (D, A, C, Folate, B_6_, B_12_) and four minerals (zinc, iron, copper and selenium) to be essential for the normal functioning of the immune system, due to the scientific evidence collected so far. In this report, an update on the evidence of the contribution of nutritional factors as immune-enhancing aspects, factors that could reduce their bioavailability, and the role of the optimal status of these nutrients within the COVID-19 pandemic context was carried out. First, a non-systematic review of the current state of knowledge regarding the impact of an optimal nutritional status of these nutrients on the proper functioning of the immune system as well as their potential role in COVID-19 prevention/treatment was carried out by searching for available scientific evidence in PubMed and LitCovid databases. Second, a compilation from published sources and an analysis of nutritional data from 10 European countries was performed, and the relationship between country nutritional status and epidemiological COVID-19 data (available in the Worldometers database) was evaluated following an ecological study design. Furthermore, the potential effect of genetics was considered through the selection of genetic variants previously identified in Genome-Wide Association studies (GWAs) as influencing the nutritional status of these 10 considered nutrients. Therefore, access to genetic information in accessible databases (1000genomes, by Ensembl) of individuals from European populations enabled an approximation that countries might present a greater risk of suboptimal status of the nutrients studied. Results from the review approach show the importance of maintaining a correct nutritional status of these 10 nutrients analyzed for the health of the immune system, highlighting the importance of Vitamin D and iron in the context of COVID-19. Besides, the ecological study demonstrates that intake levels of relevant micronutrients—especially Vitamins D, C, B_12_, and iron—are inversely associated with higher COVID-19 incidence and/or mortality, particularly in populations genetically predisposed to show lower micronutrient status. In conclusion, nutrigenetic data provided by joint assessment of 10 essential nutrients for the functioning of the immune system and of the genetic factors that can limit their bioavailability can be a fundamental tool to help strengthen the immune system of individuals and prepare populations to fight against infectious diseases such as COVID-19.

## 1. Introduction

The current COVID-19 pandemic caused by SARS-CoV-2 is reaching unexpected limits worldwide. In the first six months after the first flares were detected in China in December 2019, more than 10 million infections have officially been recorded—surely higher if real cases could be counted—with around 5% of fatal cases (between 1 and 13% recognized according to country), and the pandemic continues in crescendo (see the updated information in [1]). Consequently, factors that can condition the proper functioning of the immune system are coming into the spotlight and will increase in the coming months, when new waves can be expected. As no specific treatment or vaccine against the disease is available yet, new knowledge of other possibilities to prevent and/or improve overall disease management of COVID-19 becomes more pressing. Complementary to drug treatments and vaccines, nutritional and lifestyle factors must be considered as factors that can enhance the immune system.

Bearing in mind the fact that our organism is in constant fight against infections through the immune system, its maintenance at an optimal level of functionality is a central objective more than ever, due to the current circumstances. Thus, the relevance of optimal nutritional status for immunity must become essential. Accordingly, there is increasing knowledge as to how food, nutritional habits, and some other lifestyle aspects are essential to keeping the immune system working properly [2]. In this regard, the optimal nutritional status of some essential specific micronutrients, including vitamins and minerals, stands out above the rest of the nutrients (without playing other nutrients down) because of their contrasted potential beneficial health effects, even beyond the essential requirements classically considered in nutrition. According to the scientific information available, the competent scientific Panel of the European Food Safety Authority (EFSA) determined that the following six vitamins are important for the healthy maintenance of the immune system: D [3,4], C [3,4], A (and ß-carotene) [3], and B-group vitamins (especially B_6_, Folate, and B_12_) [5]. Consequently, the EFSA Panel concluded that there is definitive evidence of the cause–effect relationship between the daily intake of these vitamins and the normal, healthy functioning of our immune system. Meanwhile, a similar accreditation corresponds to four essential minerals: Zinc [6], Copper [7,8], iron [9], and selenium [10] are considered to be necessary for the optimal working of the immune system by the EFSA.

However, this does not mean that other nutrients, foods, or even commercial preparations with specific labelling attributing them health properties in the immune field are not also relevant, but they have not provided EFSA with sufficient scientific evidence to demonstrate this. In fact, many of the health claims that were on the market have been rejected. Consequently, and always staying within the context of balanced diets and a healthy lifestyle, it is convenient to ensure the optimal intake of these 10 micronutrients that are supported by very rigorous scientific evidence as having an essential role in the proper working of the immune system. Besides, some factors could interfere in the relationship between nutrient intake and actual nutritional status, including environmental factors, food-matrix components, and, above all, genetic features.

In this context, the main aims of this report focus on (1) identifying those nutrients scientifically supported to promote the optimal functioning of the immune system; (2) analyzing their nutritional status in European countries and studying their relationship with epidemiological COVID-19 indicators; and (3) describing genetic (and other) factors that compromise suboptimal nutritional status for the abovementioned specific nutrients. Therefore, nutritional (and nutrigenetic) features of 10 European countries (Spain, Belgium, Italy, the United Kingdom, Portugal, France, the Netherlands, Germany, Denmark, and Finland) were characterized from open databases and published studies, and contextualized within the current coronavirus pandemic framework. Thus, the relationship of identified nutritional parameters with epidemiological indicators of COVID-19 (incidence, mortality, and relative mortality) in the selected countries was analyzed.

## 2. Background

### 2.1. The European Framework of Essential Micronutrient Requirements

All healthy and balanced diets must aim to provide adequate amounts of the different nutrients to fulfil individual requirements. In this regard, dietary reference values (DRVs)—also called Dietary Reference Intakes (or DRIs)—have been set to largely cover nutritional needs mainly depending on age and gender. DRVs are defined by the EFSA as a general term for a set of nutrient reference values that include population reference intakes (PRI), adequate intakes (AI), and Tolerable Upper Intake Level limits (UL). PRIs are used to describe the distribution of requirements in a population. These give the intake of a nutrient that meets the daily needs of, respectively, half or most (97.5%) of the people in the population. AI is defined as the average level of intake of a nutrient that is assumed to be adequate, based on studies or scientific observations. UL expresses the maximum amount of a nutrient that can be safely consumed over a long period of time.

In some cases, physiological status (such as pregnancy or old age) or physical activity level are factors that are also taken into account. However, even though the PRI has been defined to mathematically cover the requirements of 97.5% of individuals, a segment of the population, including the small percentage that is not covered by the PRI, fail to satisfy their nutritional requirements with the habitual intake.

In addition, DRVs are intended for healthy people and, as such, may not satisfy nutrient demands associated with altered metabolic status. Type 2 diabetes is accompanied by increased metabolic demand of micronutrients to compensate fluid losses and reduced plasma levels. Furthermore, appropriate food choice, or even supplementation, to provide increased supply of certain micronutrients (mainly Zinc, Magnesium, Vitamin C, and B-group vitamins) is able to improve glycemic control and/or exert antioxidant activity [11,12]. Concerning obesity, a negative association between body mass index (BMI) and intake of trace elements has been identified in young obese adults [13]. In addition, obese individuals have low levels of both fat- and water-soluble vitamins, as compared to non-obese. Since increased adipose tissue mass leads to low-grade inflammation, vitamin deficiencies could exacerbate this status [14].

Regarding immunity, fulfillment of DRVs for the 10 micronutrients considered by the EFSA panel as essential for healthy maintenance of the immune system is becoming more essential than ever in order to tackle infectious diseases in the context of the current pandemic. Therefore, these 10 essential micronutrients and their DRVs established by the EFSA are listed in Table 1.

### 2.2. Factors Affecting Bioavailability of Immune Related Micronutrients

Following current dietary guidelines for immune-related micronutrients is not necessarily accompanied by correct nutritional status. An uncertain number of factors could impact on the relationship between intake and actual nutritional status. Against this backdrop, suboptimal status or marginal deficit may appear in association with the presence of specific genetic variants, environmental conditions (such as pollution), or anti-nutrient interactions in food mixtures.

#### 2.2.1. Genetic Factors

**Figure 1:**
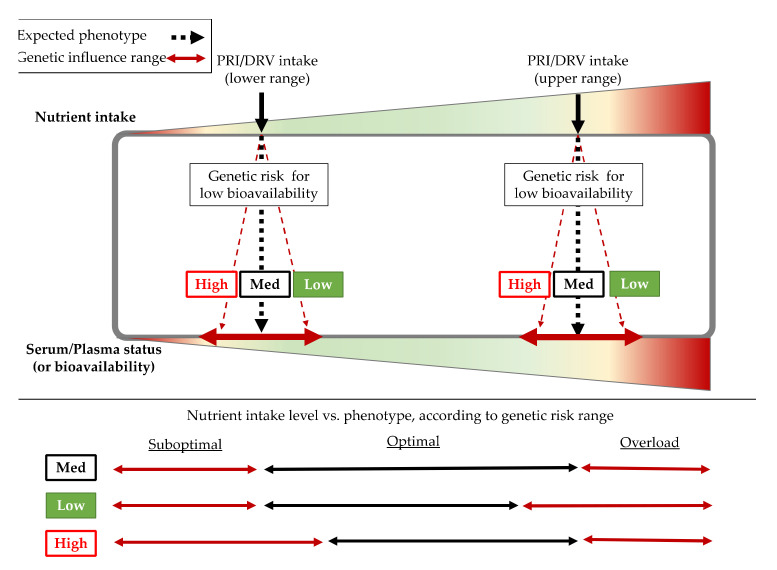
Genetic influence on the micronutrient intake-plasma level relationship. Abbreviations: PRI (Population Reference Intake); DRV (Dietary Reference Values); Med (medium genetic risk).

Genetic variants have been identified in Genome-Wide Association (GWA) studies, or association studies carried out in large cohorts, which influence different bioavailability or altered plasma/serum levels of the most relevant nutritional compounds for the immune system. DRVs or PRIs are standardized values that are based on reference nutrient intake amounts for healthy populations [32] but are based on recommendations for large populations without considering individual particularities. Therefore, different people keeping the same intake amount of a specific nutrient (even coinciding with PRIs) can trigger a spectrum of bioavailability depending on the genetic load of each individual, where each organism will manage the nutrient more or less efficiently (Figure 1). In this scenario, people with a genetic predisposition to low (specific) nutrient bioavailability would be at higher risk of non-optimal status for this nutrient. In the context of immune system requirements, genetic variants that could influence bioavailability of nutrients associated with optimal working of the immune system should be considered so as to ensure proper immunity to deal with infections. Thus, some of the most relevant Single Nucleotide Polymorphisms (SNPs, the most frequent kind of genetic variants in humans) influencing the status of the 10 micronutrients whose positive impact on the immune system is supported by the EFSA (Table 1) are described and analyzed in the context of some European countries in further sections.

#### 2.2.2. Air Pollution, Food Mixture Interaction, and Other Factors

Although not considered here, other factors such as air pollution and food mixture interactions may affect nutritional-related immunity through various mechanisms. In particular, air pollution, although still scarcely studied, may have a negative impact on vitamin D status, as active vitamin D is synthesized in the skin by exposure to ultraviolet B (UVB) radiation, and air pollution is a key determinant of the amount of UVB reaching human skin. Thus, for adults aged 18–64 years, the adequate intake (AI) for vitamin D has been set at 15 µg/day in order to achieve a serum concentration of 50 nmol/L, aiming to cover conditions with minimal cutaneous vitamin D synthesis [33].

Meanwhile, in food mixtures, specific anti-nutrient compounds can kidnap some essential micronutrients, thereby reducing their bioavailability [34]. For instance, it is well documented that the phytate content of plants, cereals, and legumes may compromise the bioavailability of minerals and this might depend on the use of fertilizers, environmental and climatic growing conditions. Concerning iron, not only phytate, but other dietary ingredients may influence its bioavailability by acting either as inhibitors (phytate and some polyphenols) or enhancers (ascorbic acid). However, the bioavailability of iron from European vegetarian diets has not been found to substantially differ from mixed diets; a PRI for adult and elderly people has been set at 11 mg/day [35], and these intake levels are achieved regardless of following vegan or vegetarian diets, or being predominantly meat or fish eaters [36].

## 3. Materials and Methods

### 3.1. Information Sources

This report focuses on the evidence of the effect on the immune system of 10 nutrients endorsed by the EFSA. In this report, the current state of affairs is updated by an unsystematic literature research performed on PubMed database. Early, more specific, data on the role of nutrients and COVID-19 were tracked in the recent LitCovid database. Furthermore, data from 10 European countries (Belgium, Denmark, Finland, France, Germany, Italy, The Netherlands, Portugal, Spain, and the United Kingdom) shown in the present report were collected from public data sources, which are detailed below.

#### 3.1.1. Literature Search Criteria for the 10 Nutrients, Immunity, and COVID-19

Once the essential nutrients for the maintenance of the immune system with scientific evidence endorsed by the EFSA were identified (Table 1), a search was carried out for each of them concerning their relationship with the immune system and COVID-19 in PubMed (https://pubmed.ncbi.nlm.nih.gov) and LitCovid (https://www.ncbi.nlm.nih.gov/research/coronavirus) databases. First, to search for current accumulated knowledge of the role of each specific nutrient in the immune system, a PubMed search was carried out as follows: for example, for vitamin C the word combination (“immunity” OR “immune system”) AND (“Vitamin C” OR “Ascorbic Acid”). Second, in order to search for the most current information on each nutrient with COVID-19, a strategy similar to the above but combining the PubMed and LitCovid databases: for instance, for Vitamin C, the combination of words (“COVID” OR “Coronavirus”) AND (“Vitamin C” OR “Ascorbic Acid”) was introduced. In each search, regular screening was carried out until 31 July and the articles that were most relevant and best suited to the purpose of this work were selected.

#### 3.1.2. COVID-19 Epidemiological Indicators

COVID-19 epidemiological indicators were consulted in Worldometers (https://www.worldometers.info/coronavirus) on 19 May 2020 in order to better represent the affection of the pandemic at an early date and thus assess the nutritional (and nutrigenetic) status of each country. From this database, COVID-19 features “Incidence” (expressed as cases per 100k people), “Deaths” (meaning mortality rate by COVID-19 expressed as number of deaths per 1M people), and “Deaths%” (meaning relative mortality rate = COVID-19 mortality% vs. cases) were collected (Table 2). Furthermore, specific nutrients whose impact on the optimal working of the immune system is supported by the EFSA were identified (Table 1) and optimal/suboptimal intake levels of each country population were assessed by compilation of data available in adults and elderly populations, in both sexes [37,38,39,40].

#### 3.1.3. Population Nutrition Data and Genetic Risk Assessment

Country nutrition data were collected as specific nutrient population intake levels and expressed as the percentage of the intake level referring to its established requirements (as 100% of PRI value) (Table 2). Moreover, nutrition data was converted to z-score to facilitate the scalation view of these data. Thus, negative values indicate that the country is below the general average, while positive percentages indicate that the country’s population% with covered requirements is above the general average.

Furthermore, relevant genetic variants influencing the status of each of the 10 micronutrients were identified using GWAs catalog tool (https://www.ebi.ac.uk/gwas) [41]. To assess genetic risk of suboptimal status of 10 specific micronutrients, individual allele risk frequency of identified genetic variants were collected from *1000 genomes* database for European populations [42]. These data included individual genetic information, as actual genotypes of desired SNPs of 396 European subjects from Spanish, Italian, Finnish, and British cohorts. According to GWAs evidence, alleles associated with lower micronutrient levels or bioavailability were identified and defined as “risk allele”. Then, a genetic score system of suboptimal risk of nutrient status was performed for each nutrient. First, a genetic risk assessment of the European population (including the data sets of the four aforementioned cohorts) was carried out by adding the total number of risk alleles (of SNPs associated with the status of the same nutrient) present in the individual sample. Second, the percentage of genetic risk was calculated as follows = actual sum of risk alleles/maximum genetic risk (that is, the number of SNP ×2 maximum risk alleles that there can be). Third, general mean and standard deviation (SD) was obtained and risk ranges of genetic risk for all European dataset was established according mean and SD. Thus, individuals were considered at low genetic risk if individual genetic risk% < [general mean − general SD]; high risk, if individual genetic risk% was > [general mean + general SD]; or medium risk if individual genetic risk% was between [general mean − general SD] and [general mean + general SD], including threshold values. Fourth, percentage of population at low, medium, and high genetic risk for each micronutrient was calculated by specific countries and statistical differences between countries were assessed by Chi-Square test using Finland as the reference of all comparisons.

#### 3.1.4. Correlation Map Performance

The correlation map shows Pearson correlations between nutritional factors and epidemiological indicators. Maps have been performed using R Software Package *corrplot*, following the guidelines of Statistical Tools for High-throughput data analysis (STHDA) [43]. Pearson correlation *p*-values were obtained with SPSS v21 (SPSS, Chicago, IL, USA).

## 4. Association between 10 Critical Micronutrients and Prevalence of COVID-19 in Europe

**Figure 2:**
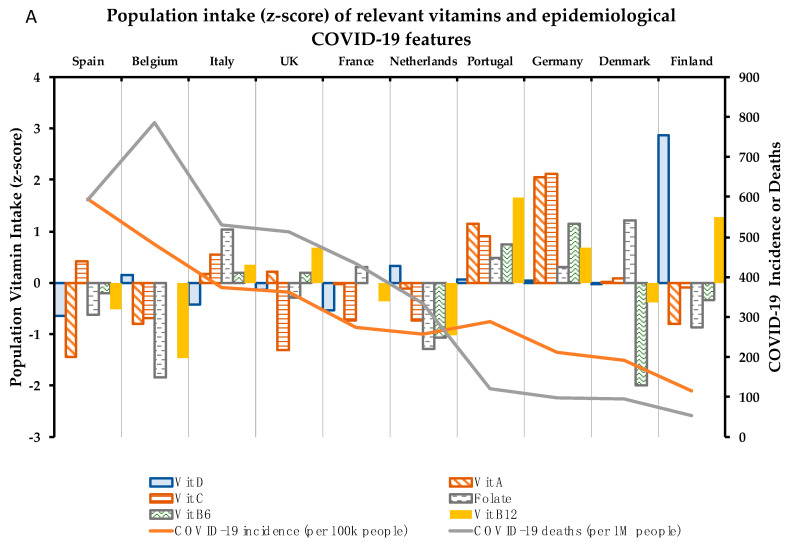

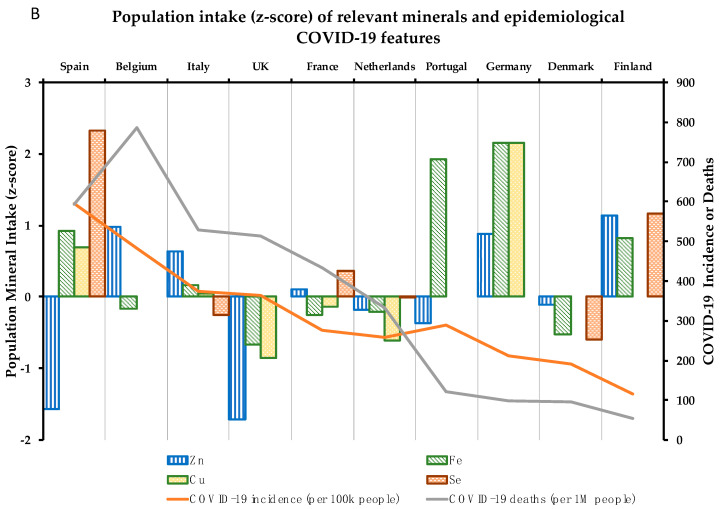
Population intake of relevant immune system Vitamins (**A**) and Minerals (**B**) in European countries and their relationship with COVID-19 Incidence (number of COVID-19 cases per 100 k people) and Deaths (mortality rate per 1 M people). Nutritional data are shown as z-score of population with vitamin or mineral requirements fulfilled and referenced to the whole data of 10 countries analyzed. Negative values indicate that the country is generally below the general median of the percentage coverage of the population nutrition requirements of the countries analyzed, whereas positive values indicate that the country population% with covered requirements is above the general median.

Concerning the 10 micronutrients with a supported role in the immune system, compilation of data available in adults and elderly target populations, in both sexes [37,38,39,40], in a number of European countries was carried out. A country-by-country analysis of the intake of the 10 micronutrients was performed (Figure 2). The effect of each identified micronutrient on the health of the immune system and their relationship of specific (sub) optimal intake with epidemiological COVID-19 parameters by country are analyzed below:

### 4.1. Vitamin D

Vitamin D plays a key role in modulating the immune system, so much so that its suboptimal or deficient consumption of vitamin D is associated with various conditions related to a malfunction of the immune system and dysregulations in inflammatory status [44]. The vitamin D receptor is expressed in most cell types of the immune system, including dendritic cells, B and T lymphocytes, monocytes, and macrophages, among others [44]. Therefore, the correct functioning of the immune system will depend largely on the correct bioavailability of vitamin D by these cells [44].

DRVs of Vitamin D are consistent along population groups and are currently based on population AI and UL [33]. Thus, AIs for male and female (including lactating and pregnant) adults are set at 15 µg/day and UL at 100 µg/day (Table 1). Despite these recommendations, there is a widespread deficit of vitamin D in European adults, particularly among the elderly, which may be associated with a combination of factors such as reduced dermal production, decreased sun exposure, and reduced food intake. Surprisingly, some northern countries, such as Finland and Iceland, have estimated intakes of 11 µg/day, higher than the intake in most European countries which ranges between 2–8 µg/day; and over 90% of older adults have a daily intake less than 10 µg [45]. This could be particularly relevant in the progression of the SARS-CoV-2 pandemic. Thus, Vitamin D also reduces the risk of microbial and viral infections by a number of mechanisms involving the maintenance of cellular physical barriers and enhancement of both natural and adaptive cellular immunity [46]. Furthermore, Vitamin D can improve cellular immunity by reducing the cytokine storm induced by the innate immune system observed in patients severely affected by COVID-19 [47], and Vitamin D supplementation could reduce the production of pro-inflammatory cytokines, increase the expression of anti-inflammatory ones, and enhance the expression of genes related to the antioxidant system [46].

The implication of Vitamin D population status in COVID-19 epidemiological factors has been checked in recent studies: negative correlations between circulating levels of Vitamin D and COVID-19 incidence were found retrospectively in a European population in two time-separated studies [48,49]; Alipio et al. significantly found lower 25-hydroxyvitamin D in most severe cases of COVID-19 in South Asian populations [50]. Furthermore, vitamin D status is associated with a 15% reduction in severe COVID-19 cases in the elderly, according to results from a multicenter study conducted in hospitals around the world based on 5000 patients up to 80 years old [51].

The biological plausibility of these findings can be explained by the ability of Vitamin D to regulate the production of proinflammatory cytokines through the inhibition of renin-angiotensin system (RAS) activity [52]; although this micronutrient paradoxically increases ACE2 in vitro expression [53], the clear immunomodulatory effects of vitamin D appear to be beneficial in preventing or treating COVID-19 [54]. Bearing in mind the above commented results, and considering that Vitamin D deficiency is a worldwide issue [55], the need for vitamin D supplements is a prominent topic in COVID-19 pandemic times, especially in the most vulnerable population groups [54].

**Figure 3:**
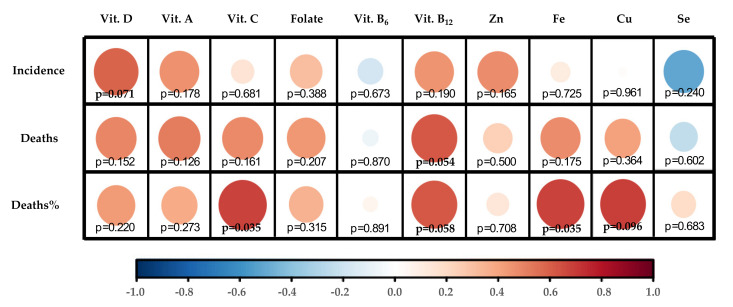
Pearson correlogram of COVID-19 epidemiological parameters: Incidence, Deaths (mortality rate), and Deaths% (relative mortality rate), and immune system micronutrient suboptimal intake. The size and color intensity of the spot indicate the degree of correlation between two parameters. The color red indicates a positive correlation, whereas blue indicates a negative correlation. Pearson correlation *p*-values (*p*) are indicated below each dot and *p* < 0.1 are highlighted in bold.

Indeed, data from European countries show general nutritional deficiency of vitamin D. Thus, only Finland (+2.9) and the Netherlands (+0.3) display z-score values of individuals with great positive vitamin D requirements reached in a European context (Figure 2A). However, these data mean that population intake levels of Vitamin D in Finland and the Netherlands only reach 62.7% and 27.6 vs. the goal of recommendations (Table 2). In contrast, Spain (14.1%), France (15.7%), and Italy (17.0%) are at the top of the negative part (Table 2), representing the countries analyzed with the highest population% with vitamin D deficiency (Figure 2). Thus, intake data showed a positive correlation between individuals% with suboptimal intake of vitamin D and COVID-19 incidence (*p* = 0.071) (Figure 3). In this scenario, Spain and Italy show the worst vitamin D intake situation coinciding with high incidence. On the other hand, Finland is at the opposite end of this relationship, with better vitamin D intake profile and better COVID-19 epidemiological indicators. Despite this, there is still some discrepancy with regard to the optimal state of vitamin D as an independent protective factor for contracting COVID-19 [56]. Possibly, the highest risk of suffering from this disease and/or greater severity would be due to a combination of related factors, such as vitamin D hypovitaminosis, which is seen in individuals with obesity or diabetes [57]. In addition, genetic factors can influence vitamin D status.

GWA studies have offered many insights into the modulating role of genetic variants on the bioavailability of vitamin D [58]. Thus, there are SNPs that influence vitamin D levels. SNPs in GC gene, which codes for the Vitamin D-binding protein are associated with differential levels of circulating protein. Thus, rs7041 C allele in GC (more common in Caucasian populations and encoding an aspartic acid for 432 aminoacidic residue) is associated with lower levels of plasma 25-Hydroxyvitamin D (specifically in African-American and Afro-Caribbean ethnics) in a trans-ethnic GWA study [17] and higher levels of Vitamin D-binding protein in Europeans [16]. Furthermore, C allele of rs1155563 (another variant in GC but not linked to rs7041) is associated with serum levels of 25-Hydroxyvitamin D in a European population [18]. Hence, GC genetic variants are a well-defined factor to be taken into account in personalized nutritional guidelines in order to guarantee a correct supply of vitamin D to maintain a healthy immune system. Furthermore, rs12785878 in NADSYN1 (coding for nicotinamide adenine dinucleotide, a key coenzyme in redox metabolic reactions) has been linked to insufficient Vitamin D [19], with the C allele (less common in European populations) associated with lower serum vitamin D levels [59]. In fact, rs7041 (GC) allelic distribution is significantly different in the Finnish population, with more frequency of the C risk allele (72.7%) than the other countries analyzed (63.1–65.9% of C allele frequency); on the other hand, the British population shows more C risk-associated allele (28%) of rs1155563 (GC) compared to Finland (20.7%; *p* = 0.031); besides, Finland is also the population with the highest frequency of C allele of rs12785878 (NADSYN1) with allelic distributions significantly different from other populations analyzed (Figure S1A). Altogether, Finland (16% of population at high risk of low Vitamin D levels and only 6% at low risk) and Spain (12% of population at high risk and 10% at low risk) show a different distribution regarding risk ranges compared to the United Kingdom (8% at high risk and 16% at low) and Italy (5% at high risk and 20% at low) (Table 3).

Analyzing intake data and potential effect of genetic variants, it can be concluded that Finland has a relative genetic deficiency of vitamin D but this is compensated with optimal intakes that could reverse the genetic risk of deficiency; meanwhile, Spain shows the high genetic risk of deficiency for this vitamin, but this risk is not compensated by intake (it is the country analyzed with the lowest population% meeting vitamin D requirements). Thus, Spain is the country whose vitamin D intake needs greater adjustment. Consequently, suboptimal Vitamin D intake is strongly associated with COVID-19 incidence as well as Deaths and Deaths% by COVID-19 (Figure 3).

### 4.2. Vitamin A

Vitamin A can be obtained from animal sources in a pre-formed form (different forms of retinol) or from non-animal sources in its precursor form (pro-vitamin A carotenoids) [60]. Most carotenoids possess immunomodulatory activities in humans and animals: these compounds are related to the correct production and cytotoxic activity of lymphocyte subsets, stimulate the release of certain cytokines, or have the ability of neutrophil/macrophage phagocytosis [61,62]. Deficiency of this vitamin is associated with many serious immune disorders in mucosal features and impaired adaptive response [63]. In addition, negative effects can be heightened in case of obesity [64]. Thus, nutritional deficiency in this vitamin is associated with an increased risk of measles and diarrhea [65], while its supplementation is associated with lower morbidity and mortality derived from infectious diseases (measles-related pneumonia, HIV infection, and malaria) [66,67].

In addition, vitamin A and some other retinoids also show important immunomodulatory properties, including the ability to increase the efficiency of actions of type 1 interferons (IFN-I), an important antiviral cytokine released by the innate immune system against viral infections [68]. However, coronaviruses similar to SARS-CoV-2 can suppress the host IFN-I-based antiviral response as part of their infection mechanism [68]. Therefore, retinoids have recently been considered as adjuvants to increase IFN-I-mediated antiviral responses, their effect could even be tested in combination with antiviral drugs currently tested in preclinical studies of SARS-CoV-2, especially those based on IFN-I mediated response [69,70].

Vitamin A PRIs for adults are set at 650 μg RE/day for women and 750 μg RE/day for men while the UL are set at 3000 RE/day (Table 1), but PRIs differ depending on other factors, including age, sex, and physiological features: requirements and UL are lower for children and infants and PRIs, but not UL, are higher for pregnant (700 μg RE/day) and for lactating women (1300 μg RE/day) [71]. However, the intake data by country show that Spain is once again the country with the lowest population meeting nutritional requirements for vitamin A (77.2%, Table 2) in comparison with the other countries analyzed. Following Spain, Belgium and Finland are located in the second lowest position by number of population (Figure 2), but both reaching population requirements (with values over 100%) (Table 2). Meanwhile, Germany (256%) and Portugal (210%) show the best numbers in this context. In this case, with the exception of Finland, countries with suboptimal Vitamin A status are correlated (although not significantly) with their COVID-19 incidence and mortality (Figure 3).

GWA studies revealed a number of genetic variants associated with differential levels of carotenoids [20,72,73] and retinoids in human plasma [74]. Circulating carotenoid levels are inversely associated with a risk of various disorders, such as metabolic syndrome [60,62], while there is some controversy regarding this effect and the circulating levels of retinol [60]. In this context, the most relevant and characterized genetic variant associated with carotenoid plasma concentration is rs6564851, with BCO1 SNP as the one whose biological mechanism has been elucidated in more detail and has been associated with differential levels of various plasma carotenoids [20]. This SNP is found near the gene that encodes 15,15′-monooxygenase 1 (BCO1), an enzyme expressed in the small intestine, liver, and other tissues such as the adipocyte, which catalyzes the isometric cleavage of β-carotene and other provitamin A carotenoids into retinol molecules (two in the case of ß-carotene) [75,76]. G allele carriers of rs6564851 show lower activity of BCO1 than T carriers [77], explaining 1.9% of the variance in plasma β-carotene, and, to a lesser extent, higher circulating levels of other pro-Vitamin A carotenoids [20]. However, rs6564851 has not been found to be associated with retinol levels [20], the beneficial/harmful effects of low/high concentrations of which on health remain unclear to date [60]. Thus, rs6564851 may be the most characterized genetic biomarker to evaluate genetic risk of Vitamin A deficient/suboptimal status.

In this context, Finland is the country with the least (40.4%) T risk allele presence among its citizens, followed by the United Kingdom (47.3%), Spain (57.9%), and Italy (59.8%) (Figure S1B). Thus, Finland may be the population with the least genetic risk (just 12% of their population within high risk range) of low Vitamin A status among the European countries analyzed (Table 3). Taking intake and genetic data together, Spain must be the country with the greatest need to adjust population intake of Vitamin A. Finland may make up the low intake shown because of its lower genetic risk of deficiency.

### 4.3. Vitamin C

Vitamin C has a key antioxidant role, so its optimal nutritional contribution is related to less oxidative damage [78] and its supplementation is associated with a lower incidence of specific infections [79]. Furthermore, studies performed on chicken embryos showed Vitamin C treatment induces resistance against avian coronavirus [80]. Despite no proven benefit associated with Vitamin C supplementation once common cold symptoms have already started, its supplementation to prevent or fight COVID-19 has been suggested [81].

Beyond supplementation, intravenous (IV) administration of Vitamin C at high doses could be associated with lower mortality in patients with severe sepsis, according to the results of a meta-analysis [82]. Specifically, infusion of 15 g/day of Vitamin C for 4 days reduces the mortality of patients with sepsis and severe acute respiratory failure [83]. With this knowledge, early results based on reports of small groups of COVID-19 patients who received IV Vitamin C as part of common treatment improved their clinical outcomes [84]; hence, a new controlled trial focused on testing the effect of IV Vitamin C (at a dose of 24 g/day for 7 days) in patients with COVID-19 was recently launched [85]. The physiological plausibility of this treatment might be based on its antioxidant and anti-inflammatory benefits, which could reduce the typical cytokine storm triggered in acute respiratory distress syndrome [86].

Concerning dietary status of Vitamin C, DRVs also depend on population groups. For adults, PRIs are set at 95 mg/day in women and 110 mg/day for men (Table 1), while in young ages it ranges between 20–100 mg/day, depending on sex and age. In the same trend as Vitamin A, pregnant and lactating women need higher intakes (105 and 155 mg/day, respectively) [87]. No upper limit of vitamin C has been established so far. Correspondingly, Vitamin C optimal status must play an important role in the proper working of the immune system. In this regard, countries such as the UK (75.3%), France (86.9%), Netherlands (86.6%), and Belgium (87.8%) do not reach optimal dietary intake of Vitamin C (Table 2). Contrarily, Germany stands out for its level of Vitamin C intake (143.9%) (Table 2) in comparison with other countries (Figure 2). Despite Vitamin C suboptimal intake correlating weakly with COVID-19 incidence, it correlates stalwartly with deaths% (*p* = 0.035) (Figure 3), which could suggest a positive effect to fight infection once the individual has already been infected with SARS-CoV-2.

There is no scientific evidence associating plasma vitamin C levels with genetic variants in GWAs, although the association between rs33972313 and vitamin C has been replicated in large cohorts [21,88]. This SNP is located in the SLC23A1 gene, coding for one of the sodium-dependent transporters involved in vitamin C absorption and body distribution [89]. Hence, CT and TT genotype carriers (minors in European populations), are associated with 5.6% and 11.9% decreased Vitamin C plasma levels, respectively [21]. Taking this SNP as a genetic risk reference, the Spanish population may show a lower genetic risk to Vitamin C suboptimal status (with <1% of T risk allele frequency; *p* = 0.001 compared to Finland) (Figure S1C and Table 3). This increased genetic risk of Vitamin C deficiency coincides with the low vitamin C nutritional status in the UK population, which could further aggravate the suboptimal status effect for this micronutrient.

### 4.4. Folate

Folate (or Vitamin B_9_) plays essential roles in one-carbon mediated metabolism, amino acid and nucleic acid metabolism, and DNA methylation [90]. Furthermore, Folate is also crucial for optimal Th-1 mediated immune response and proper antibody production [91]. Thus, suboptimal levels of Folate intake may trigger imbalances in T and NK cell mediated immune responses [92] and decrease amount of antibody production [91]. Although scientific evidence is still scarce, Folate could play a role in COVID-19 management: it may participate in furin inhibition activity (a convertase involved in SARS-CoV-2 spike protein cleavage) and consequently prevent this necessary step for the virus’ access into host cells [93]. Furthermore, homocysteine metabolic pathway disruption by COVID-19 infection has recently been proposed as one of the mechanisms that lead infected cells to death [94]. Therefore, Folate, as well as Vitamins B_6_ and B_12_, should be considered in the treatment of SARS CoV-2 infections to suppress these complications.

Regarding Folate DRVs, PRIs and UL are set at 330 μg of dietary Folate equivalent (DFE)/day and 1000 μg/day, respectively, for female and male adults (Table 1). However, PRI values are increased for lactating women (500 μg DFE/day) and PRI and UL values are decreased in children and infants [95]. Focusing on the European framework of Folate intake levels, there is a certain homogeneity in relation to the number of individuals with fulfilled recommendations in the European countries analyzed. Therefore, Belgium (63.0%) and the Netherlands (68.5%) show poor intake levels (Table 2), being the countries with the smallest number of individuals with Folate requirements covered (Figure 2). As regards the relationship between Folate intake and COVID-19 incidence and mortality, Folate suboptimal status correlated positively but not significantly with these epidemiological parameters, although less potently than vitamins D, A, and C (Figure 3).

In this context, the most relevant SNP is surely rs1801133 (C677T) located in the MTHFR gene, which codes for methylenetetrahydroFolate reductase enzyme. This SNP causes an Alanine 222 to valine exchange [96] and has been associated with differential levels of Folate [22] and Homocysteine [97] in plasma in GWAs. Specifically, the presence of the less common allele (T) reduces MTHFR enzyme activity to 35% (for each copy of the allele), resulting in higher levels of homocysteine and lower levels of plasma Folate [22,98]. Furthermore, the prevalence of the MTHFR 677TT genotype varies depending on the population, and it has been observed that the administration of folic acid is able to smooth the differences attributed to the genotype. These particulars, added to the fact that unmetabolized folic acid in plasma is associated with a reduction in the cytotoxicity of natural killer cells [92], make this SNP a marker to take into account in order to adapt nutritional guidelines for proper functioning of the immune system. Thus, Italy (46.7%, *p* < 0.001) and Spain (44.4%, *p* < 0.001) show a higher occurrence of T allele risk than Finland (27.3%), while the British population (32.4%) shows a similar frequency to the Finnish (Figure S1D). Therefore, taking C677T SNP as the marker to evaluate genetic predisposition for low-Folate status, Italian and Spanish populations would show greater risk of that (Table 3) and nutritional/clinical strategies should be considered to cover this issue.

### 4.5. Vitamin B_6_

It has been described that correct contribution of dietary Vitamin B_6_ (also called Pyridoxine) is essential for maintaining cytotoxic activity of NK cells [99], appropriate lymphocyte development, and B-cell antibody production [100]. Otherwise, suboptimal consumption of this vitamin is associated with lower concentrations of circulating lymphocytes [99], impaired lymphocyte maturation, and decreased antibody-based responses [101]. In the same trend as folate, the correct supply of Vitamin B_6_ (and B_12_) in patients affected by COVID-19 has been proposed as part of the disease treatment [94], even by supplementation formulas, in an attempt to regulate the disruption of cellular metabolism of the homocysteine pathway caused by the SARS-CoV-2 infection [94].

Vitamin B_6_ established requirements are 1.6 mg/day for women and 1.7 mg/day for men, with UL of 25 mg/day for both sexes (Table 1). Following the trend of the above analyzed vitamins, requirement values are increased in the case of being pregnant or lactating (1.8 and 1.7 mg/day, respectively) and PRI and UL are decreased for children and infants [102]. Data on Vitamin B_6_ intake displayed in Table 2 show that most of the countries analyzed are at a similar population intake level% with requirements covered (around or over 100% value), with the exception of Denmark (86.2%). With the z-score applied, Denmark followed by the Netherlands proved to be the countries with the lowest Vitamin B_6_ intake levels, and Germany, the country with the highest intake levels (Figure 2). Despite this, there is certain homogeneity regarding Vitamin B_6_ intake levels among the countries analyzed. Furthermore, no clear relationship with epidemiologic COVID-19 indicators has been found for this micronutrient (Figure 3).

Regarding genetic variants affecting Vitamin B_6_ requirements, the presence of each C allele of rs4654748, located in the Neuroblastoma breakpoint family, member 3 (NBPF3) gene, is associated with decreased Vitamin B_6_ levels, 1.45 ng/mL in case of heterozygosity or 2.90 ng/mL (around 17 nmol/L) in case of C homozygosity, compared to TT genotype carriers [23]. Differences between two people with opposite genotypes may explain a huge 57% of the variation in Vitamin B_6_ plasma levels and, consequently, trigger personalized intake requirements. Despite no differences in rs4654748 genotype distribution found between the European populations analyzed (Figure S1E), a significant part of the whole population analyzed (20.6–29.9%) must be at high genetic risk of Vitamin B_6_ suboptimal status (corresponding with carriers of CC genotype), meaning that approximately a quarter of the population requires special nutritional demands (Table 3). Despite this, following the trend of intake levels of this B-group vitamin, no differences were found between the countries analyzed regarding genetic influence on poor Vitamin B_6_ status. Consequently, epidemiological COVID-19 differences by country could not be attributed to Vitamin B_6_ nutrigenetic status.

### 4.6. Vitamin B_12_

Sufficient Vitamin B_12_ intake is also essential for antibody production and clonal expansion [91,99]. Thus, its deficiency is related to a lower concentration of circulating lymphocytes [7] and altered antibody-based responses [63].

Vitamin B_12_ has recently been proposed as a potential therapy against COVID-19 [103]. This evidence was gathered through in silico approaches aiming to find potentially effective molecular models against SARS-CoV-2, by integrating genetic sequences and the relative information of previous treatments against the SARS-CoV and MERS viruses. In brief, this virtual screening was based on the identification of antiviral compounds, vitamins, antimicrobials, and other systemically acting drugs that can act against two proteases necessary for the processing and release of translated non-structural proteins from coronaviruses, the so-called protease type 3-C (M-pro) and a papain-like protease (Plpro) [104]. According to the results of this study, Vitamin B_12_ would be the fourth compound with the highest docking score against M-pro, only behind Chromocarb, Ribavirin, and Telbivudine drugs [103].

In addition, based on the fact that SARS CoV-2 infection is related to an aggravation of the cellular metabolism and the homocysteine pathway causing severe complications from COVID-19, the correct supply of Vitamin B_12_ and the aforementioned Folate and Vitamin B_6_ must be crucial for COVID-19 patients [94]. Therefore, this evidence supports the relevance of this vitamin in the immune system and to fight COVID-19.

Vitamin B_12_ intake requirements are established by the EFSA at 4 μg/day for the general group of adults (Table 1), 4.5 μg/day in pregnancy, and 5 μg/day for lactating women. Vitamin B_12_ requirements range between 1.5–4 µg/day among children and infant age-groups [105]. Nutritional data about Vitamin B_12_ show quite a similar intake among populations (Table 2) with homogeneity and values above the requirements. However, the contextualization of the intake levels% in the COVID-19 pandemic European framework displays that some of the countries least affected by the pandemic show the highest levels of Vitamin B_12_ intake (as is the case of Portugal and Finland). On the other hand, Belgium and Spain, with intakes below the median (negative z-scores) are among the countries most affected by the coronavirus (Figure 2). Altogether this may explain that the suboptimal intake of this micronutrient is strongly correlated with the epidemiological parameter’s “deaths” (*p* = 0.054) and “deaths% vs. cases” (*p* = 0.058) (Figure 3).

The impact of genetic variants on plasma Vitamin B_12_ levels has been shown in a European population-based GWA study [23]. In this study, rs11254363 (CUBN), rs526934 (TCN1), and rs602662 (FUT2) were associated with differential levels of this vitamin depending on the allelic load [23,24]. It is widely accepted that 203–542 pg/mL (150–400 pmol/L) Vitamin B_12_ serum ranges are considered optimal [105]. Hence, carriers of risk alleles of the aforementioned SNPs may show 21.49–49.77 pg/mL decreased levels of Vitamin B_12_ (depending on the SNP affected), which could mean 10–25% higher risk of lowering the optimum concentration threshold, even when following recommended intakes. This should be specifically relevant in Finnish and British populaces, where (taking into account the frequencies of 3 SNPs shown in Table 1) around 14% and 9% of the population are ranged at high risk of low Vitamin B_12_ levels, and only 5% and 4% are ranged at low risk (Table 3), respectively. Individually, rs11254363 (CUBN) A risk allele is more frequent in Finnish (77.3%) and British (78.6%) populations; rs526934 (TCN1) G risk allele is more frequent in the British population (33.5%); while rs602662 (FUT2) G risk allele is more common in the Finnish population (Figure S1F).

### 4.7. Zinc

Zinc optimal status is essential for the proper operation of the immune system, since this trace element exerts antioxidant effects, protecting against ROS and reactive species [106]. In addition, zinc influences the antioxidant activity of some proteins, among several other relevant functions [99]. Thus, zinc, in optimal levels, acts as an anti-inflammatory compound by helping to optimize immune responses at the same time as reducing risk of infection [107]. On the other hand, zinc deficiency is associated with immune dysfunction and the consequent susceptibility to infectious diseases [108]. Moreover, its deficiency is also associated with impaired NK cell activity and neutrophil/macrophage phagocytosis activity, T-cell mediated antibody response, and abnormalities in complement activity [99].

Regarding the coronavirus framework, previous scientific evidence supports that Zinc optimal intake, or appropriate supplementation, should be considered part of the strategy to reduce COVID-19 effects (recently reviewed in [107]). Furthermore, in vitro approaches have pointed out that zinc can inhibit SARS-CoV-1 replication by blocking its polymerase activity [109].

Directly related to COVID-19, recent evidence supports a direct connection between zinc status and the disease caused by the new coronavirus [110,111] This importance is based on the necessary participation of zinc-dependent viral enzymes to carry out the infection process. In addition, drugs tested against SARS-CoV-2, such as chloroquine, cause increases in the intracellular concentration of this micronutrient. Consequently, zinc treatments (alone or as an adjuvant for other drugs) are currently being tested in clinical trials for COVID-19 [110,112].

As an example of zinc treatment not as an adjuvant to other drugs, there is a recent pre-proof medical report based on four hospitalized patients clinically diagnosed with COVID-19 treated with oral high-doses of zinc salts (15–23 mg/day) who showed significant improvement in the disease after one day of treatment, suggesting that high-dose zinc therapy may play a role in clinical recovery [113].

Taking this evidence together, it can be concluded that the importance of meeting the nutritional requirements of zinc can be extracted. Guidelines to follow these recommendations are a little bit more complex compared to the other nutrients analyzed, since the influence of phytate as an antinutrient for zinc absorption is well documented by the EFSA [114]. Thus, zinc PRIs in adults are conditioned by the intake of phytate. On the other hand, the UL for this micronutrient is set at 25 mg/day by the EFSA (Table 1).

In the same trend as Vitamins B_6_ and B_12_, zinc requirements are generally covered among the European countries analyzed, with just the UK (79.4%) and Spain (81.2%) being far from reaching recommended population intake levels (Table 2). Z-scores calculated in the European context show that the UK and Spain have greater suboptimal zinc intake levels compared with the rest of countries, with Finland and Germany displaying the best values (Figure 2B). Despite this, populations with suboptimal zinc intake levels are not significantly correlated with higher COVID-19 incidence rates (Figure 3).

In a European population-based GWA study, SNPs located in the CA1 gene (coding for Carbonic Anhydrase-1, a zinc-dependent enzyme involved in important procedures, including maintenance of blood pH homeostasis [115]); PPCDC (coding for phosphopantothenoylcysteine decarboxylase, involved in the biosynthesis of coenzyme-A from pantothenic acid [116]); and NBDY (coding for Negative Regulator of P-Body Association, a micropeptide involved in the formation of the P body and the processing of mRNAs [117]) were associated with differential zinc levels in a European population [25]. Thus, carriers of C alleles for each rs2120019 (PPCDC), rs1532423 (CA1), and rs4826508 (NBDY) SNPs are associated with lower levels of serum zinc [25]. Allele distribution of rs4826508 is the only one of the mentioned zinc associated SNPs to shows differences among the European populations analyzed. Thus, C risk allele is significantly (*p* < 0.001) more frequent in British (18.7%), Italian (38.8%) and Spanish (37.4%) than in Finnish (14.6%) populations (Figure S1G). This means that, when considering the three SNPs, the countries with the highest percentage of people at high genetic risk of zinc deficiency are Italy and Spain (Table 3). These genetic differences observed in the Spanish population, together with low population zinc intake levels (Table 2) could possibly indicate a potential weak point of the common immune system in the Spanish population due to the lack of zinc.

### 4.8. Iron

Iron participates in several immune processes, and is an essential component for some enzymes involved in crucial activities of immune cells [118,119]. Due to its structure, iron also plays an important role as a mediator of oxidative stress situations (acting as a redox catalyst) and also exerts powerful antimicrobial effects by forming highly toxic hydroxyl radicals for infection agents [118,119]. Therefore, deficient or suboptimal levels of iron are associated with decreased killer efficiency of NK cells and lymphocytes as well as with compromised cytokine production [118,119]. Therefore, both iron uptake disturbances and metabolism are implicated in virulence of airway hospital-acquired infection and chronic respiratory infections [120]. In contrast, excessive iron levels can generate harmful cellular toxicity [118], so their serum levels must be well regulated. In this regard, it is important to monitor the intake of this mineral and follow the established recommendations. Thus, iron PRIs are set at 16 mg/day for lactating, pregnant and premenopausal women, and 11 mg/day for postmenopausal women and men populations [35] (Table 1). Although EFSA has not established the UL for iron yet, it could be considered that the tolerable upper intake level for iron in adults is set at 45 mg/day, based on gastrointestinal adverse effect levels [121].

Regarding the COVID-19 context, iron metabolism is clearly disrupted in SARS-CoV-2 infected patients, especially in subjects hardest hit by the disease [122], consequently anemia [123] or hyperferritinemia are typical manifestations of hospitalized COVID-19 patients. Therefore, optimal iron status may be crucial for better disease prognosis. So, a retrospective study based on 50 hospitalized Chinese subjects with confirmed COVID-19, demonstrated that 90% of these subjects (45) showed abnormally low serum iron concentrations (<7.8 μM) and the severity of the disease negatively correlated with serum iron concentrations [124]. For this reason, populations with lower iron status could be more prone to suffer a *mild to severe* (or critical) symptomatology of COVID-19 and the fact of monitoring patient iron levels has been proposed as a potential early marker to predict COVID-19 severity and mortality [124]. Despite this, blind supplementation of patients with iron can be a double-edged sword, since excess serum iron and hyperferritinemia in those affected by COVID-19 are also associated with increased inflammation and tissue fibrosis [125]. In this context, iron chelation can be used as a supportive treatment for COVID-19 related iron overload, which has also shown antiviral and antifibrotic proprieties [125], in an attempt to reduce disease severity and improve clinical outcomes [126].

Compliance with iron requirements is key to promoting personal health and avoiding deregulation; lower plasma iron levels are associated with decreased lung function, and excessive tissue iron content is related to high pulmonary inflammation [127]. Data concerning iron intake in European countries show that the UK is located in the bottom ranking of intake levels (86.9%) (Table 2). Figure 2B shows suboptimal iron intake by the British population in contrast to German (129.6%) and Portuguese (126.1%) populations which have higher intake levels of iron (see values in Table 2) and are also located below in the ranking of Deaths (Germany, 97; Portugal 122 per 1M inhabitants) and Deaths% vs. cases (Germany, 4.6%; Portugal 4.2%) (Table 2) and iron intake levels could have played a role in boosting the immunity of their citizens. By contrast, the UK is the worst placed in this ranking, coinciding with higher mortality (513) and Death% vs. cases (14.1%) by COVID-19 (Table 2). Thus, iron suboptimal nutritional status of countries is strongly correlated (*p* = 0.035) with Death% vs. cases (Figure 3).

In this context, correct individual bioavailability of iron emerges as a basic requirement for optimal functioning of immune system components. Variants in genes that code for key proteins for the iron metabolism, such as Human Homeostatic iron Regulator Protein (HFE), Transferrin (TF), or Transferrin Receptor 2 (TFR2), have been associated with interindividual variability of this micronutrient bioavailability. Thus, the most frequent variant (G) of rs1800562 (also called C282Y), located in the HFE gene, is associated with decreased levels of iron, Ferritin, plasma Hemoglobin, higher Transferrin levels and lower Transferrin saturation [27]. Therefore, G allele may be considered a potential risk factor for iron deficiency and even anemia. Despite this, “G to A” exchange in rs1800562 (affecting around 5–10% in many Caucasian populations) entails Cys-282 to Tyr change [128], and is associated with higher circulating iron levels [26], increased risk of hemochromatosis, liver disease, rheumatoid arthritis, osteoarthritis, and diabetes, especially in men and postmenopausal women [129]. Thus, this SNP would have a double tessitura depending on the genotype of which it is a carrier. Therefore, its analysis appears to be essential for personalized nutritional/clinical planning. In the same sense, the major rs1799945 allele (C) in European populations (SNP also located in the HFE gene, although in linkage equilibrium with rs1800562) is likewise associated with decreased serum iron levels (4.95 µg/dL lower per copy of C allele) [28,29] as well as lower plasma Hemoglobin concentration [130]. While the minor allele (G), which entails the His 63 residues exchange for Asp, is associated with increased risk of hypertension [131]. Whereas no differences are shown between European populations concerning rs1800562 (HFE), the major rs1799945 C allele (associated with lower iron bioavailability) is less frequent in all the countries analyzed (74.8–82.2%), compared with Finnish (88.9%) (Figure S1H).

The other relevant SNPs affecting optimal iron bioavailability are rs3811647 and rs7385804, respectively located in the TF gene and its receptor (TFR2). The major allele of rs3811647 (G) is associated with decreased ferritin levels [29], lower total iron-binding and unsaturated iron-binding capacities [26]; while the minor allele (A) of rs7385804 has been associated with lower serum iron concentrations and transferrin saturation [27]. Consequently, these last two SNPs would also explain the great variability in factors for iron bioavailability in addition to, but independent of, the abovementioned HFE genetic variants. Within the European framework, rs3811647 (TF) the low-iron associated allele is more frequent in Finnish than in British and Spanish populations, while the low-iron risk allele of rs7385804 (TFR2) is more frequent in the Finnish population compared with the rest of the countries analyzed (Figure S1H). All in all, Finland would be the country with the highest frequency (37%) of population with a high risk of showing low iron status and fewer people with low genetic risk (7%) (Table 3). Consequently, the Finnish population could be more prone to developing an iron deficiency and iron dependent immune functions, compromised due to these genetic features of their population.

### 4.9. Copper

Copper is a trace element that plays a key role in optimal performance of relevant components of the immune system, such as NK cells, macrophages, neutrophils, and monocytes [132]. Its deficiency has been related to less effective immune responses against infections and increased virulence [91,99]. Furthermore, suboptimal copper intake (even without reaching critical deficiency) is associated with decreased T-cell proliferation and abnormalities in macrophage phagocytosis [133]. On the other hand, excessive intake of this mineral would be associated with negative effects on the immune system [99].

Furthermore, in vitro studies show that copper ions block a fundamental protein for SARS-CoV-1 replication [134]. Hence, copper has antiviral properties acting at two levels: enhancing the components of the immune system to fight against infections and by direct contact with virus [135].

Consistent with this evidence, it has been hypothesized that the optimal state of plasma copper levels can increase both innate and adaptive immunity, even exerting an effect as a preventive and therapeutic factor against COVID-19 [136]. Following these lines, a recent review identified copper as a candidate to study its effects in combination with other drugs, such as N-acetylcysteine, Colchicine, or Remdesivir, as strategic treatment for COVID-19 treatment [137].

Copper nutritional requirements are also based on IA, with 1.3 mg/day for adult women (increased to 1.5 mg/day when lactating or pregnant) and 1.5 mg/day for adult men, and the UL are set at 5 mg/day [138] (Table 1). Attending the importance to reach the intake recommendations of this micronutrient, copper is a nutritional component whose intake levels must be accurately monitored. The nutritional data analyzed reveals that the UK is in suboptimal intake of copper (70.2%), while Germany has the best nutritional indicator for this micronutrient (157.8%), considering the European framework (Table 2). Following a similar way as in the case of iron, but to a lesser extent, suboptimal nutritional status of copper is negatively correlated with Death% vs. cases (*p* = 0.096) (Figure 3).

Therefore, personal requirements, taking into account age, sex, presence of pathologies, and genetics, must be considered so as to achieve optimal nutritional status. Thus, according to the evidence in a GWA study based on a European population, the influence of the genetic variants rs2769264 and rs1175550 on the levels of copper in serum could be observed [25]. These genetic variants are found, first, in the gene that codes for Protein Selenium Binding 1, SELENBP1, and, second, in the SMIM1 (Small integral membrane protein 1) gene, coding for an integral membrane protein involved in antigen presentation [139]. In this way, carriers of the T and A allele of rs2769264 and rs1175550 would present decreased serum copper levels [25].

Regarding rs2769264 (SELENBP1), the allele related to lower copper levels is the most common in European populations, according to *1000genomes* database (Figure S1I). Despite this, British population is the only one to have a lower frequency of the T risk allele (79.7%, including heterozygotes and risk allele homozygotes) compared to the Finnish population. The other populations analyzed have a very similar profile in the allele distribution of this SNP. In the same sense, risk allele (A) of rs1175550 (SMIM1) shows pretty much the same frequency among all the populations analyzed (Figure S1I), fluctuating between 74.8 and 83.3%. Taken both SNPs together, all four countries would have a similar % of population at high genetic risk of compromised copper status, with more than a third of each population exposed (Table 3).

### 4.10. Selenium

Selenium is an important trace element involved in differentiation, proliferation, and normal functioning of many components of the innate immune system. In addition, selenoproteins (proteins that includes a selenocysteine aminoacidic residue in their sequence) have a role as antioxidants and in attenuating free radicals released during immune response. Furthermore, selenium is also crucial in the adaptive response, assisting in antibody production and development [91]. For this reason, suboptimal or deficient levels of selenium are associated with decreased cytotoxicity of NK cells, decreased antibody titers, impaired cellular immunity, and decreased response to vaccination; while its supplementation is commonly related to improvements in cellular immunity and an improved optimal immune response against viruses [99]. In fact, selenium supplementation is associated with an inhibitory effect on the development of the poliovirus and influenza [140].

Some more recent data suggest that the optimal status of selenium might be key to promoting immunity against SARS-CoV-2 infection: First, a recent retrospective population analysis found a positive correlation between the rate of COVID-19 recovered patients in 17 Chinese cities (outside of Hubei) on 18 February 2020, and the selenium status of the city’s population (previously measured by hair selenium concentration) [141]. These epidemiologic findings may be supported by the dependence of glutathione peroxidase 1 (GPX1) activity (a selenoenzyme with described antioxidant and antiviral properties [142]) on nutritional selenium status [143]. Second, selenium would act as an inhibitor of reactive oxygen species (ROS) derived from viral infection as well as the potential interaction between GPX1 and the main protease (M-pro) of SARS-CoV-2, as has recently been suggested [144]. Consequently, the optimal status of selenium has gathered strength because some pharmacological compounds with selenium in their chemical structure could inhibit SARS-CoV-2 protease [145]. Furthermore, bioinformatic approaches have provided new insights into selenium metabolism disruption induced by SARS-CoV-2 infection [146]. Furthermore, the use of selenium salts (sodium selenite) has also been proposed as a therapy to reduce the risk of blood clot formation [147] observed in patients infected by SARS-CoC-2, which is one of the important COVID-19 collateral death causes [148]. In view of the evidence mentioned above, it is logical to assume that ensuring optimal selenium status (even using supplements of selenium salts) could represent a strategy for the prevention of viral infections, including the coronavirus and associated clinical complications [147].

Intake recommendations for selenium are based on AI: 70 µg/day (85 µg/day in the case of lactating women); with UL established at 300 µg/day for both adult women and men [149] (Table 1). Data regarding selenium intake show high dispersion among countries (Figure 2B), with Spain having the highest indicator of met selenium requirements (108.2%), and Denmark being the country with the least (53.2%) (Table 2). There are only two populations above the median of the countries analyzed—Finnish and French—while the rest of the countries are below the general median (Figure 2B). Thus, the correlogram shows a non-significant inverse correlation between suboptimal status of selenium and COVID-19 incidence (Figure 3), but that may be due to the limited data available on the intake of this micronutrient and its difficult nutritional assessment.

Therefore, genetic variants related to decreased levels of selenium could be a risk factor that compromises the functionality of the immune system. In this context, individuals carrying A alleles for both rs891684 and rs17823744 SNPs are associated with decreased levels of selenium, which could compromise the bioavailability of this micronutrient to circulating immune cells. These two polymorphisms have been most strongly associated with differential levels of selenium in GWAs based on European populations, especially rs891684 located in the SLC39A11 (solute carrier family 39 member 11), a gene that codes for a zinc transporter [30]. On the other hand, rs17823744 is located in the DMGDH gene, which codes for dimethylglycine dehydrogenase (an enzyme involved in the catabolism of choline in the mitochondrial matrix) that uses adenine-flavin dinucleotide and Folate as cofactors, and this SNP is associated with differential levels of selenium in toenails [31], an alternative marker to assess the nutritional status of selenium [150].

The rs891684 (SLC39A11) frequency of risk allele in Finnish and Italian populations are quite similar (<10% of A allele) while British (15.4%, *p* > 0.001) and Spanish (14.0%; *p* = 0.022) samples showed higher A allele frequency than the Finnish population (Figure S1J). On the other hand, no differences were found between European populations for rs17823744 (DMGDH), with the presence of A allele risk fluctuating between 85.0–91.4%. Taking both SNPs into account to estimate the genetic risk of low selenium status, different distributions of population% in genetic risk range were found concerning Spanish (low: 18; medium: 74; high: 8; *p* = 0.037) and British (low: 21; medium: 67; high: 12; *p* = 0.033) compared to the Finnish population (low: 26; medium: 70; high: 4) (Table 3).

## 5. Discussion

The highest European authority in food safety, EFSA, endorsed the impact of the correct nutritional status of vitamins (D, A, C, Folate, B_6_ and B_12_) and minerals (zinc, iron, selenium and copper) on immune system health, so the scientific evidence of the beneficial effect of these micronutrients on immunity and protection against infective diseases is considered to be beyond doubt. In this report, an updated review of the specific effect of these 10 micronutrients on the immune system was carried out, focusing on the context of the COVID-19 pandemic.

Population intake levels of 10 of these specific nutrients and their compliance with country-by-country recommendations were analyzed and contrasted with epidemiological features of the SARS-CoV-2 pandemic. Observational data suggest that vitamin/mineral requirements are not fulfilled by diet in the EU and could be a limiting factor in the immune response to fight against infections.

The nutritional data analyzed, displayed in Table 2, shows that Vitamin D intake is rather deficient in all the countries (to a different extent depending on the country), with Spain, France, and Italy as the countries with the lowest intake. This scenario is repeated in the case of Folate, but without reaching such low intake levels. Suboptimal iron intake is found in 6/10 of the countries analyzed; and Vitamin C and zinc in 5/10. On the other hand, Vitamin A intake levels are only suboptimal in Spain. None of the countries analyzed revealed intake below the recommendations with regard to Vitamin B_12_. Despite the fact that selenium, copper, and Vitamin B_6_ data are missing for some countries, suboptimal consumption of selenium (6/7) and copper (5/7) can also be observed. In contrast, only two countries have intakes below the Vitamin B_6_ recommendations.

Although cross country comparison is limited by the heterogeneity of data collection protocols and dietary assessment methods, a number of previous dietary surveys have found widespread prevalence of suboptimal intakes for several micronutrients across the European region [151], ranging from 11 to 30% for copper, Folate, selenium, Vitamin B_12_, and Vitamin C [152]; and four of the eight trace minerals (i.e., selenium, zinc, Iodine, and copper) show a high prevalence of insufficiency in the elderly, which is worsened by the requirement for institutional care [40]. Thus, country specific genetic requirements have also been taken into account as a factor to more accurately point out the risk of suboptimal intake of the selected nutrients.

In this context, the European countries worst hit by the pandemic show population% with suboptimal intake of vitamins and minerals that are important to the immune system. Thus, incidence of infection by the new coronavirus and deaths caused by COVID-19 are associated with poor dietary intake of vitamins D, A, and B_12_ (Figure 3). In this scenario, Spain shows the worst data in relation to insufficient vitamin D and vitamin A intake, and is located among the top-3 of the worst vitamin B_12_ data (Figure 2A), coinciding with the country with the highest incidence of COVID-19 and the second in mortality, at the time the data analysis was performed (Table 2). In addition, Spaniards could display genetic features related to a higher risk of having lower circulating levels of Vitamins A and B_12_, for which they are deficient, which might further compromise whispered deficiencies. In the same way, the next two countries with the highest incidence of COVID-19 (after Spain and Belgium), Italy and the UK, show deficiencies in the consumption of Vitamin D and, therefore, a higher genetic risk for its deficiency, which could also be related to the high percentage of deaths with respect to COVID-19 cases. Thus, needs of vitamin D coverage seem to play a key role in maintaining the immunity of individuals [46], which would be reflected in the greater effect of the pandemic in countries with major deficiencies in its consumption. In this fashion, Vitamin D status must be monitored in well-characterized deficient populations to prevent COVID-19 incidence; and its supplementation ought to be considered, presumably in patients with deficient serum levels (i.e., levels < 10 ng/mL) in order to reduce disease morbidities and/or mortality rates [54].

Besides, a lower population% with covered needs of vitamin C, iron, and copper is related to higher relative mortality rates from COVID-19 (Figure 3). Accordingly, the UK presents a large population% with suboptimal intake for these three micronutrients; and, in addition, greater genetic risk of decreased levels of vitamin C.

Beyond the 10 essential nutrients with evidence supported by the EFSA on their role in the proper function of the immune system, there are other nutrients and bio-active compounds, the evidence of which is still scarce, but which could also boost immunity and must be considered.

In this regard, Polyunsaturated Fatty Acids (PUFA) are a bio-active group of compounds present in fish oils, algae, and other food sources [153]. Despite the fact that genetic and anthropometric factors should be taken into account to consider their benefits, omega-3 PUFAs eicosapentaenoic acid (EPA) and docosahexaenoic acid (DHA) show anti-inflammatory proprieties [154,155] and a combined adequate intake of 250 mg/day is considered necessary for the maintenance of general cardiovascular health [156]. In this context, the potential benefits (and risks) of omega-3 PUFA supplementation to COVID-19 patients were recently reviewed [157]. The authors of this last paper concluded that more randomized, controlled trials are needed to evaluate the benefits of EPA and DHA supplementation because, even though EPA and DHA might improve inflammatory resolution and recovery of COVID-19 patients, it is also plausible that the excess of these compounds could promote non-enzymatic oxidation of cell membranes and cellular damage by increased pro-oxidative cell status [157].

In addition, other essential vitamins and minerals beyond the 10 considered by the EFSA could be candidates to be considered for their role in the potential improvement of the immune system due to their antioxidant potential and as modulators of the activity of immune cells, namely Vitamin E [158] and Magnesium [159]. Moreover, other food rich bio-actives or bio-active compounds *per se* have also been proposed either as potential enhancers of the immune system or as part of COVID-19 drug treatments. For instance, it is well documented that curcumin exerts health benefits on the immune system because of its positive effects on oxidative stress, microbiota, and antibacterial or antiviral action [160]. Subsequently, this polyphenol has also been proposed recently as a potential treatment for this novel coronavirus disease, based on its hypothetical capacity to inhibit cell entry or virus encapsulation [160] or on the indirect relationship with cardiovascular outcomes, ACE2 expression, and curcumin treatment observed in animal models [161]. In the same way, the probable immunoenhancing effect of other promising phytonutrients must be considered as potential preventive nutritional treatments (or even in formula preparations), such as stilbenes [162] or quercetin [163]. Finally, it is imperative to keep watching out for the health of the microbiota by means of pre- and probiotics to boost the host’s immune system [164]. Beyond specific nutrients or bioactive compounds, the potential impact on immunity of diets that cause an elevation of ketone bodies or their exogenous administration must also be assessed. According to the conclusions of a recent review, ketone bodies are endogenous metabolites involved in cellular energy homeostasis, but they also have important signaling activities that affect immunological activity. Therefore, knowledge regarding the role of ketone bodies in the fight against respiratory viral infections and the development of intervention studies based on the administration of exogenous ketone bodies as new immunometabolic therapies for these diseases could offer potential resources to deal with the SARS-CoV-2 pandemic [165]. Finally, due to the continuous production of scientific evidence and the results of the first clinical trials, it cannot be ruled out that other compounds must also be considered as potential strengtheners of the immune system, and other elements beyond genetics must be taken into account as factors that require less bioavailability of these essential nutrients. Therefore, it is necessary to continuously review the state of the art of factors that may play a role in the prevention and fight against COVID-19.

It is important to mention that the approach analyzed here responds to an ecological study profile and the results obtained must be considered within the range of advantages and limitations of this type of approaches. On the one hand, ecological designs are frequently carried out to study the first suspicions of the negative health effects of certain conditions (in this case nutritional and genetic) in the group setting. This kind of approach presents some advantages, if the data is readily available, such as being an inexpensive and fast approach to provide an early exploration of an association.

On the other hand, the fact that ecological studies in epidemiology are characterized by their observation unit being a population group and not individuals separately, it is a limitation that could entail some biases. Thus, there is no direct evidence to show that exposures to independent factors (such as suboptimal intakes of different nutrients or genetic variants of risk to suboptimal status of specific nutrient) and the incidence or mortality from COVID-19 occur in the same people, but rather that the results are based on comparisons made in general population groups. Furthermore, the application of the ecological study approach implies that the relationships analyzed cannot be adjusted for other factors that might have an impact on the differences in the incidence and death rates from COVID-19 in these countries. Some of these confounding factors not analyzed here could be socioeconomic factors, age of individuals, levels of air pollution, incidence of respiratory and metabolic diseases in the population, and other factors.

## 6. Conclusions

In conclusion, this report considers the potential influence of the 10 essential nutrients, considered critical by the EFSA for the proper functioning of the immune system, by reviewing their potential preventive or other effects against COVID-19. In this context, it is worth noting that the countries with the worst intake profile for these micronutrients correspond to those that have received the cruelest blow from the COVID-19 pandemic. The results of this ecological study show that the suboptimal consumption of Vitamin D, Vitamin C, Vitamin B_12_, and iron is correlated with either COVID-19 incidence or mortality indicators. Moreover, the scientific evidence accumulated to date highlights the relevance of the optimal status of the 10 nutrients but, above all, it underlines the importance of Vitamin D and iron for the immune system as well as for the prevention and fight against COVID-19. Thus, the body of evidence suggests conducting epidemiological scientific studies, intervention studies, and/or in vitro approaches in order to establish and characterize the benefits of Vitamin D and iron (or even their combination) against COVID-19.

In addition, genetic factors that predispose their carrier to a suboptimal nutritional status for these specific nutrients must be taken into consideration. The analysis of genetic variants associated with lower bioavailability (or lower circulating levels) of essential micronutrients for the optimal functioning of the immune system emerges as a potential and useful tool to detect population groups predisposed to suboptimal phenotypes. Therefore, the detection of individuals with a high genetic risk of showing low levels of specific nutrients would enable the preventive application of personalized nutritional guidelines to promote, in the first instance, individual health, and, as such, improve population health.

Thus, both suboptimal intake and genetic risk of suboptimal status should be screened, and consequent nutrigenetic strategies must be put in place to help individuals to reach an optimal nutritional state and, consequently, improve the whole population’s immunity to face this pandemic.

## Figures and Tables

**Table 1:** List of nutrients with contribution to the functioning of the immune system endorsed by the European Food Safety Authority (EFSA); Dietary Reference Values (DRVs); genetic factors that affect nutritional status and risk allele that predisposes to lower status; population in which the association was proven; and bibliographic citation of the genetic association paper. Dietary Reference Intakes were consulted on EFSA DRV Finder interactive tool [15]. Zinc PRIs * co-depend on Phytate intake levels and are expressed here as the mean of 300, 600, 900, and 1200 mg/day PRI values.

Micronutrient [EFSA Cite]	EFSA DRVs	SNP Affecting Status (Gene)	Associated Trait	Risk Allele	Population Based Discovery	Cite
Vitamin D [3,4]	AI: 15 μg/day UL: 100 μg/day	rs7041 (GC)	↑ vitamin D-binding protein ↓ Serum Vitamin D **	C C	1380 European 8541 African American or Afro-Caribbean	[16,17]
		rs1155563 (GC)	↓ Serum Vitamin D	C	761 European	[18]
		rs12785878 (NADSYN1)	Vitamin D Deficiency	C	16125 European	[19]
Vitamin A (and ß-carotene) [3]	PRI: 650 (w)/50 (m) μg RE/day UL: 3000 μg RE/day	rs6564851 (BCO1)	↓ Serum Carotenoids	T	1191 European	[20]
Vitamin C [3,4]	PRI: 95 (w)/110 (m) mg/day UL: ND	rs33972313 (SLC23A1)	↓ Serum Vitamin C	A	9234 European	[21]
Folate [5]	PRI: 330 μg DFE/day UL: 1000 μg DFE /day	rs1801133 (MTHFR)	↓ Folic acid in red blood cells	T	2232 European	[22]
Vitamin B_6_ [5]	AI: 1.6 (w)/1.7 (m) mg/day UL: 25 mg/day	rs4654748 (NBPF3)	↓ Serum vitamin B_6_	C	2934 European	[23]
Vitamin B_12_ [5]	PRI: 4 μg/day UL: ND	rs11254363 (CUBN)	↓ Serum vitamin B_12_	A	2934 European	[23]
		rs526934 (TCN1)	↓ Serum vitamin B_12_	G	2934 European	
		rs602662 (FUT2)	↓ Serum vitamin B_12_	G	2934 European	
			↓ Serum vitamin B_12_	G	1001 South Asian	[24]
Zinc [6]	PRI*: 10.1 (w)/12.9 (m) mg/day UL: 25 mg/day	rs2120019 (PPCDC)	↓ Serum Zinc	C	2603 European	[25]
		rs1532423 (CA1)	↓ Serum Zinc	C	2603 European	
		rs4826508 (NBDY)	↓ Serum Zinc	C	2603 European	
**Iron** [9]	PRI: 11 (w + m)/16 (w^#^) mg/day UL: ND	rs1800562 (HFE)	↑ Unsaturated iron-binding capacity	G	679 European	[26]
			↑ Total iron-binding capacity	G	679 European	
			↓ Transferrin saturation	G	23986 European	[27]
			↑ Transferrin levels	G	23986 European	
			↓ Serum iron	G	23986 European	
			↓ Ferritin levels	G	23986 European	
			↓ Hemoglobin	G	4818 European	
		rs1799945 (HFE)	↓ Transferrin saturation	C	12375 Hispanic or Latin American	[28]
			↓ Serum iron	C	5633 European	[29]
			↓ Serum iron	C	12375 Hispanic or Latin American	[28]
			↓ Hemoglobin	C		
		rs3811647 (TF)	↓ Unsaturated iron-binding capacity	G	679 European	[26]
			↓ Total iron-binding capacity	G	679 European	
			↓ Transferrin	G	5633 European	[29]
		rs7385804 (TFR2)	↓ Serum iron	C	23986 European	[27]
**Copper** **[7,8]**	AI: 1.3 (w)/1.6 (m) mg/day UL: 5 mg/day	rs2769264 (SELENBP1)	↓ Serum copper	T	2603 European	[30]
		rs1175550 (SMIM1)	↓ Serum copper	A	2603 European	[31]
**Selenium** **[10]**	AI: 70 µg/day UL: 300 µg/day	rs891684 (SLC39A11)	↓ Serum Selenium	A	1203 European	[25]
		rs17823744 (DMGDH)	↓ Toenail Selenium	A	4162 European	

Abbreviations: SNP (Single Nucleotide Polymorphism); AI (Average Intake); PRI (Population Reference Intake); UL (Tolerable Upper Intake Level); RE (Retinol Equivalents); ND (Not defined, as data were inadequate to derive a value); DFE (Dietary Folate Equivalent); * (w) and (m) mean recommendations for women and males, respectively; and (w^#^) means for premenopausal women; ** Variant influence on vitamin D serum levels not been confirmed in European ethnic.

**Table 2:** COVID-19 epidemiological parameters and Vitamin and Mineral population intake levels (% vs. requirements) per country vs. recommended intake values.

	COVID-19 Parameters			Vitamin Intake (% vs. Requirements)						Mineral Intake (% vs. Requirements)			
**Country**	I	M	D%	D	A	C	Folate	B_6_	B_12_	Zn	Fe	Cu	Se
**Spain**	595.0	593.0	10.0	14.1	77.2	109.9	74.9	112.2	128.1	81.2	110.9	115.4	108.2
**Belgium**	481.6	786.0	16.3	25.0	110.0	87.8	63.0	ND	111.3	112.4	94.5	ND	ND
**Italy**	373.5	529.0	14.2	17.0	160.0	112.5	91.1	117.9	143.8	108.2	99.5	96.5	59.6
**UK**	363.2	513.0	14.1	21.0	162.5	75.3	78.1	117.8	149.6	79.4	86.9	70.2	64.3
**Portugal**	288.5	122.0	4.2	23.9	210.0	119.4	85.8	125.8	166.9	95.9	126.1	ND	ND
**France**	275.7	433.0	15.7	15.7	150.0	86.9	84.0	ND	130.8	101.7	93.3	90.8	71.2
**Netherlands**	258.3	334.0	12.9	27.6	144.9	86.6	68.5	99.7	119.1	98.2	93.9	77.0	64.2
**Germany**	211.9	97.0	4.6	23.5	256.0	143.9	83.9	131.4	149.4	111.2	129.6	157.8	ND
**Denmark**	190.7	95.0	5.0	22.5	152.0	103.2	92.8	86.2	130.6	99.1	89.2	ND	53.2
**Finland**	115.5	54.0	4.7	62.7	110.0	99.8	72.5	110.3	160.0	114.3	109.5	95.0	86.4

Abbreviations: I (Incidence); M (Mortality rate); D% (Death% vs. cases); Zn (Zinc); Fe (iron); Cu (Copper); Se (Selenium); and ND (no data shown for this item).

**Table 3:** Genetic risk of lower status of 10 relevant Vitamins and Minerals for proper immune system function. Population% at low, medium, and high risk of low micronutrient status are shown calculated from individual SNP data for European Population (available in *1000genomes* database) as described in the Materials and Methods. Statistical assessment of differences in population% at risk ranges for each country was carried out by Chi-Square with Finland used as reference country. *p*-values in bold means *p* < 0.05.

	Finnish	British	Italian	Spanish
Vitamin D				
Low	6.1	16.5	19.6	10.3
Medium	77.8	75.8	75.7	77.6
High	16.2	7.7	4.7	12.1
χ^2^ (*p*-value)	Ref.	**0.000**	**0.000**	0.217
Vitamin A				
Low	31.3	28.6	13.1	18.7
Medium	56.6	48.4	54.2	46.7
High	12.1	23.1	32.7	34.6
χ^2^ (*p*-value)	Ref.	**0.032**	**0.000**	**0.000**
Vitamin C				
Low	96.0	90.1	95.3	99.1
Medium	4.0	7.7	4.7	0.9
High	0.0	2.2	0.0	0.0
χ^2^ (*p*-value)	Ref.	0.146	0.764	**0.001**
Folate				
Low	51.5	44.0	30.8	28.0
Medium	42.4	47.3	44.9	55.1
High	6.1	8.8	24.3	16.8
χ^2^ (*p*-value)	Ref.	0.267	**0.000**	**0.000**
Vitamin B_6_				
Low	21.2	18.7	29.0	23.4
Medium	54.5	51.6	50.5	46.7
High	24.2	29.7	20.6	29.9
χ^2^ (*p*-value)	Ref.	0.473	0.216	0.275
Vitamin B_12_				
Low	5.1	4.4	15.9	13.1
Medium	80.8	86.8	78.5	77.6
High	14.1	8.8	5.6	9.3
χ^2^ (*p*-value)	Ref.	0.152	**0.000**	**0.023**
Zinc				
Low	29.3	31.9	21.5	21.5
Medium	62.6	59.3	55.1	59.8
High	8.1	8.8	23.4	18.7
χ^2^ (*p*-value)	Ref.	0.800	**0.001**	**0.011**
iron				
Low	7.1	17.6	17.8	19.6
Medium	55.6	69.2	61.7	59.8
High	37.4	13.2	20.6	20.6
χ^2^ (*p*-value)	Ref.	**0.000**	**0.000**	**0.000**
Copper				
Low	17.2	15.4	19.6	15.9
Medium	38.4	45.1	38.3	44.9
High	44.4	39.6	42.1	39.3
χ^2^ (*p*-value)	Ref.	0.407	0.801	0.422
Selenium				
Low	26.3	20.9	24.3	17.8
Medium	69.7	67.0	69.2	73.8
High	4.0	12.1	6.5	8.4
χ^2^ (*p*-value)	Ref.	**0.033**	0.571	**0.037**

## Supplementary Materials

The following are available online at https://www.mdpi.com/2072-6643/12/9/2738/s1, Supplementary Figure S1. Country genotype frequencies of Single Nucleotide Polymorphisms (SNPs) involved in specific Vitamins and Minerals nutritional status.
