# Supplementary material for: Current State of Evidence: Influence of Nutritional and Nutrigenetic Factors on Immunity in the COVID-19 Pandemic Framework
Source: Nutrients. 2020 Sep 8;12(9):2738. doi: 10.3390/nu12092738 (PMC7551697; doi:10.3390/nu12092738)
Supplement: Supplementary file 1 [file nutrients-12-02738-s001.pdf]

Supplementary Figure 1. Country genotype frequencies of Single Nucleotide Polymorphisms (SNPs) involved in specific Vitamins and Minerals nutritional status[1].

A. SNP frequencies in European populations that may influence Vitamin D status

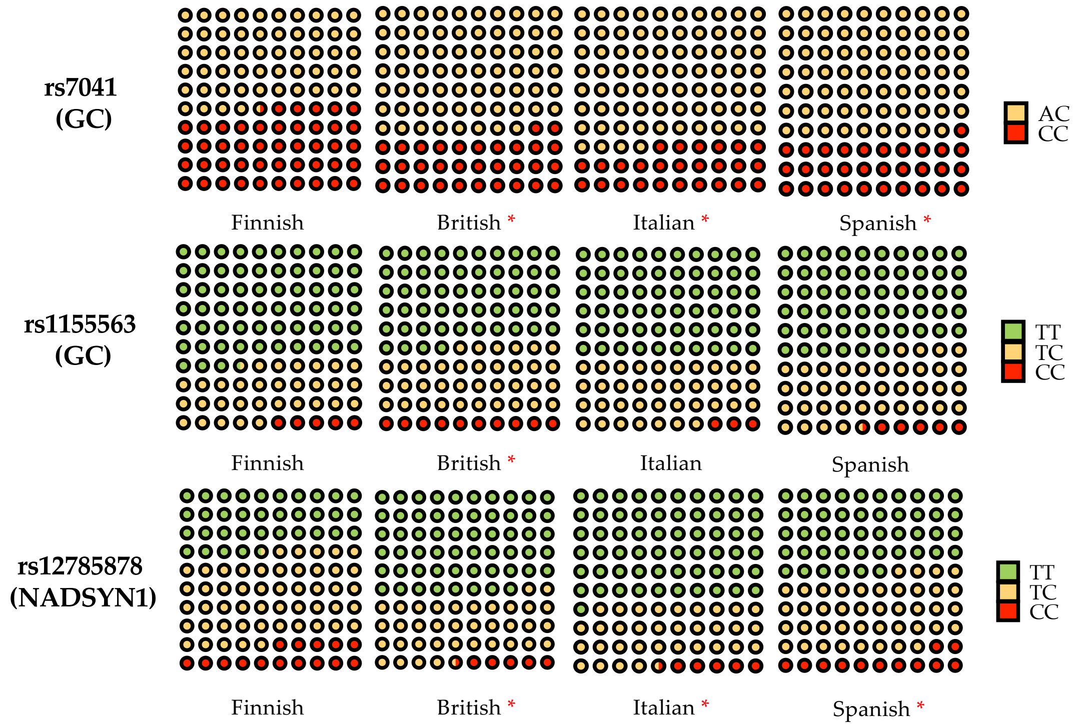

B. SNP frequencies in European populations that may influence Vitamin A status

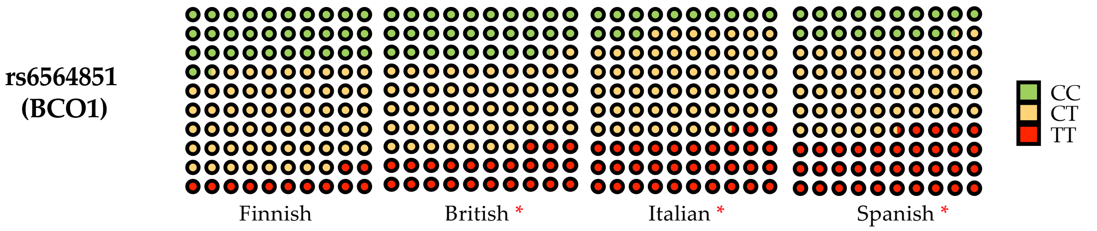

C. SNP frequencies in European populations that may influence Vitamin C status

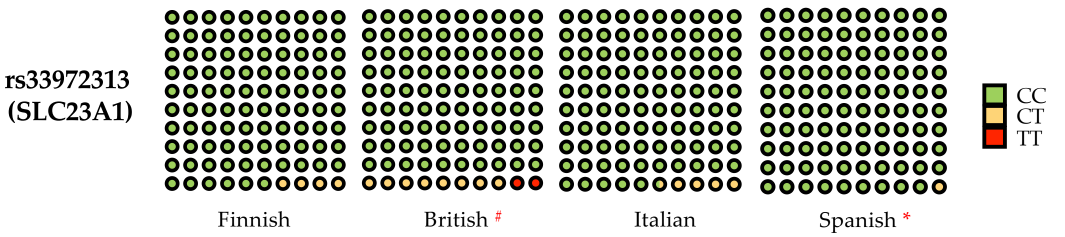

D. SNP frequencies in European populations that may influence Folate status

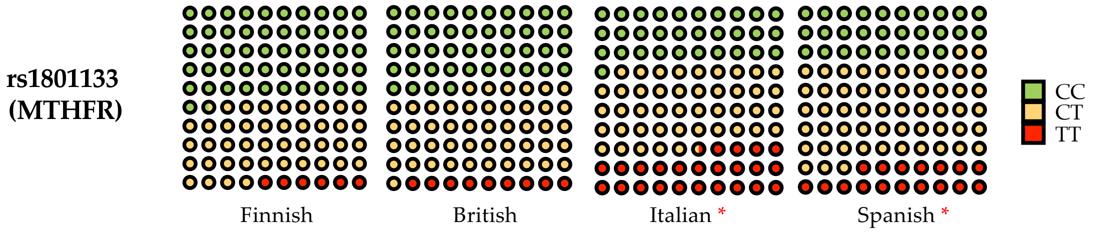

E. SNP frequencies in European populations that may influence Vitamin B_6 status

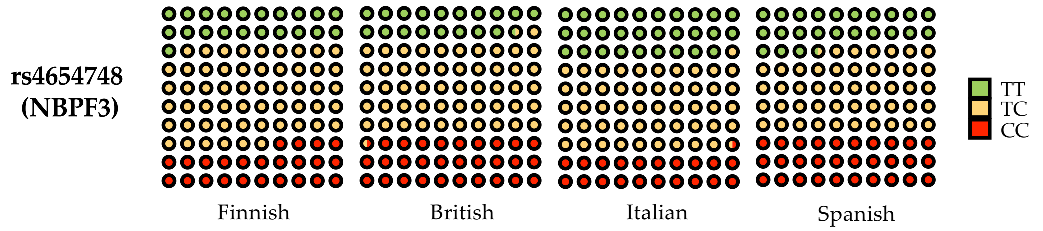

F. SNP frequencies in European populations that may influence Vitamin B_{12} status

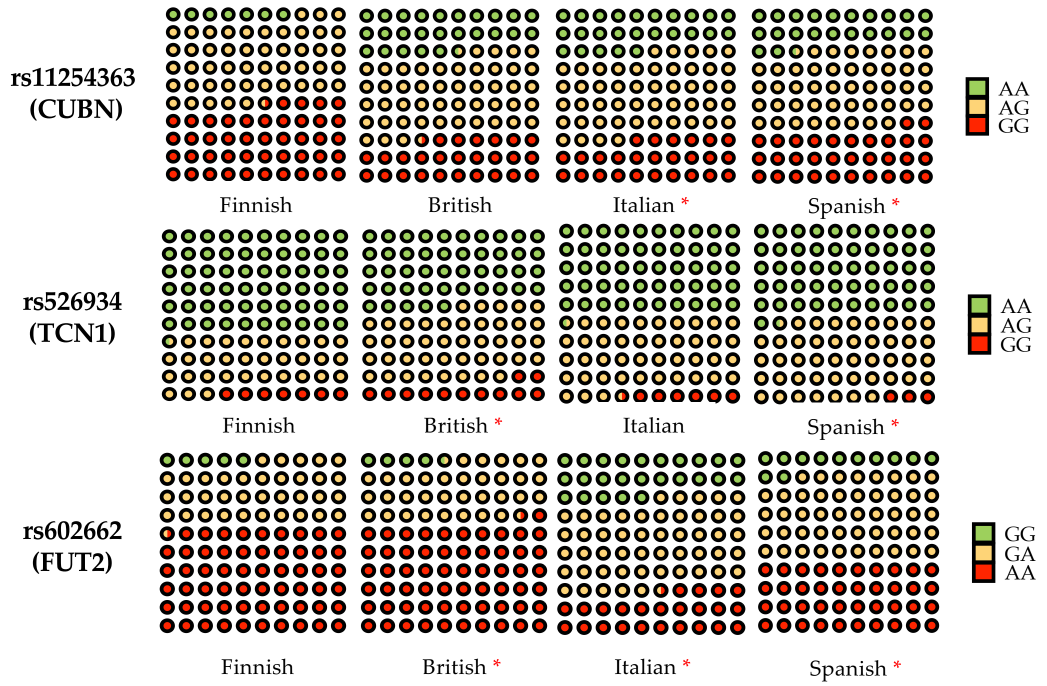

G. SNP frequencies in European populations that may influence Zinc status

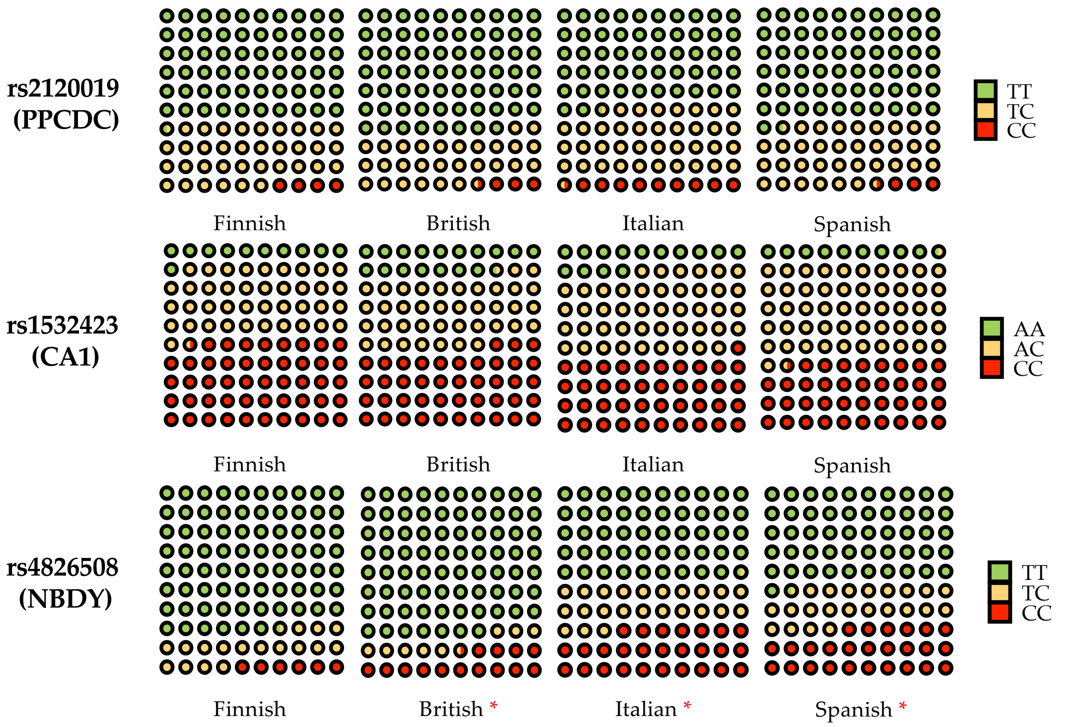

H. SNP frequencies in European populations that may influence Iron status

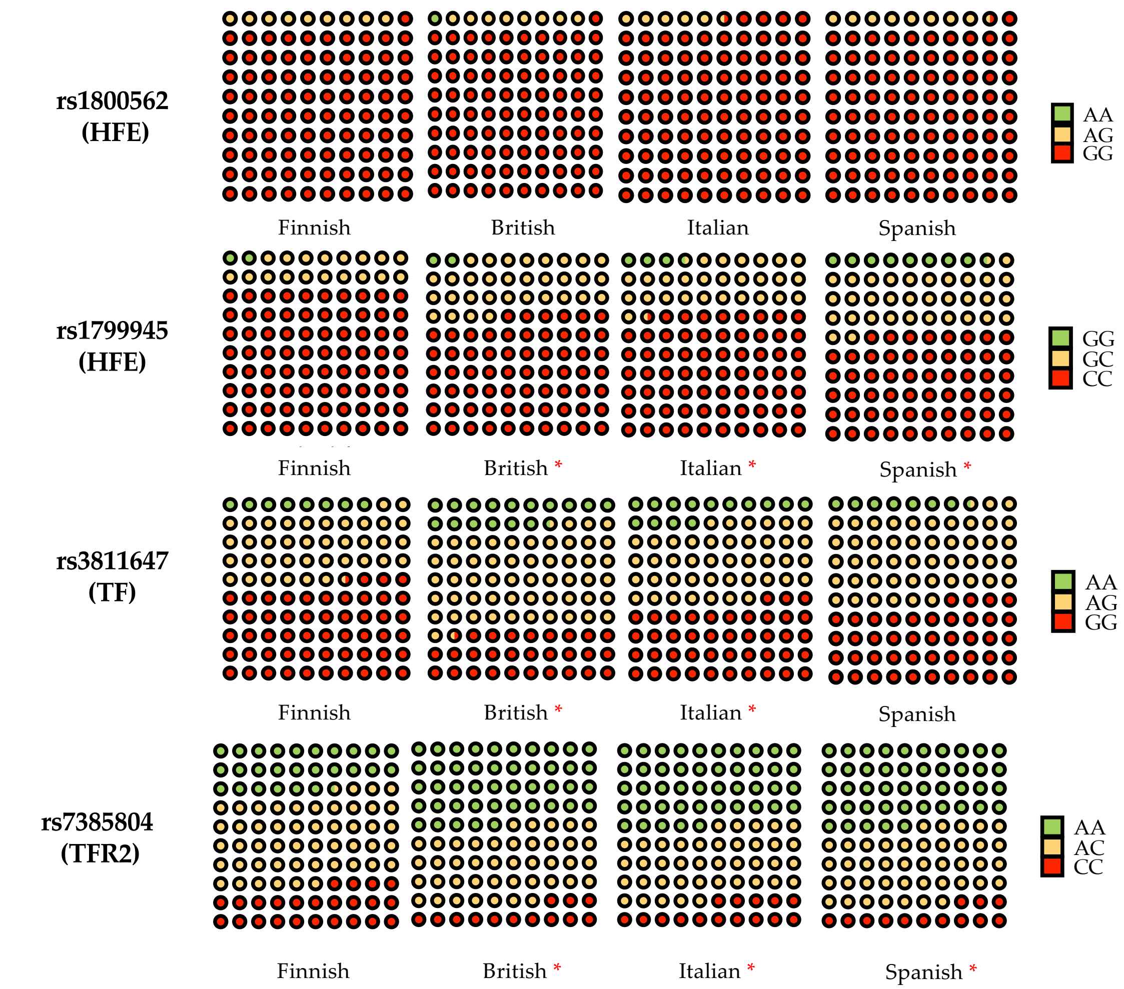

I. SNP frequencies in European populations that may influence Copper status

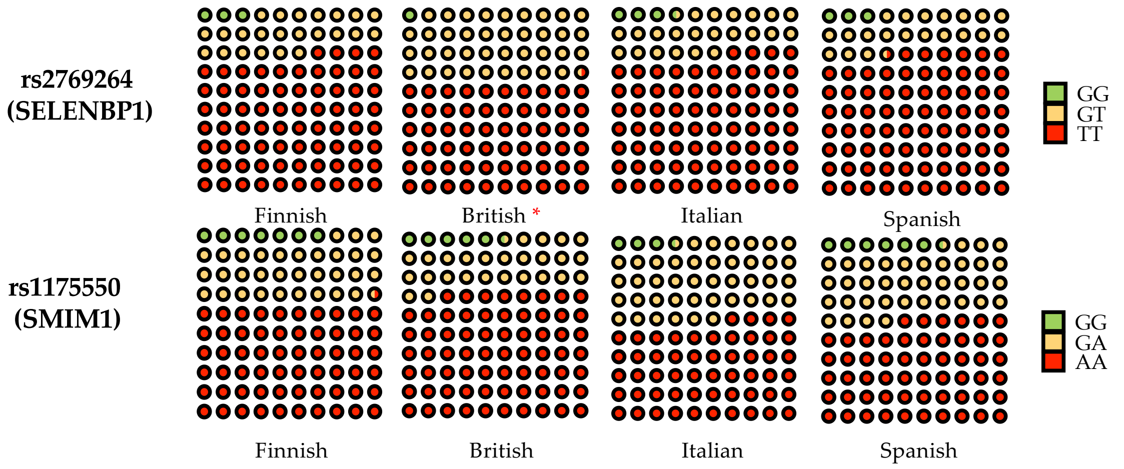

J. SNP frequencies in European populations that may influence Selenium status

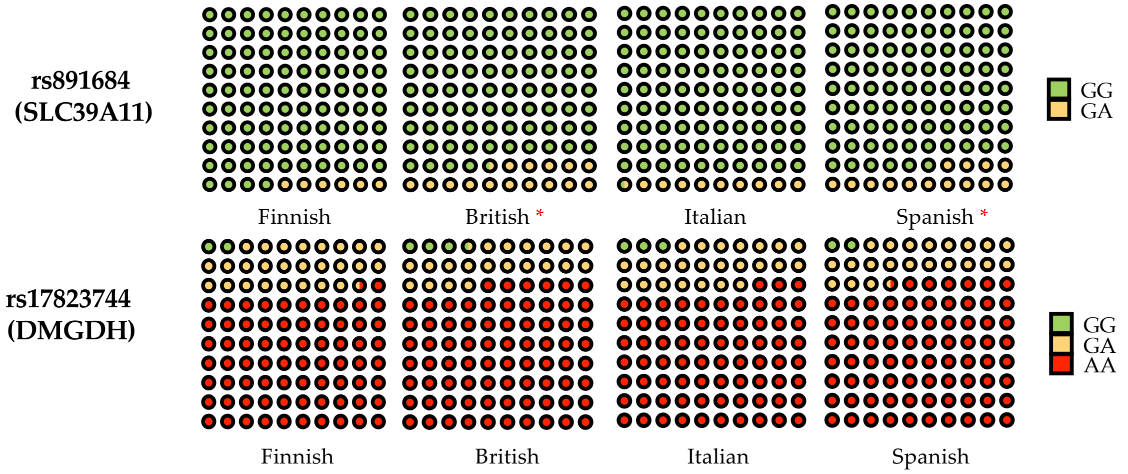

[1]Frequency (%) of genotype combinations of each SNPs involved in Vitamin/Mineral status by country. SNPs are classified in blocks according to the nutrient on which they can affect their bioavailability or circulating levels. For each SNP, the percentage of occurrence of each possible allelic combination is represented in the indicated population: green dots represent the percentage of expected individuals carrying the genotype no associated to genetic risk for low levels or suboptimal status of the indicated nutrient; yellow dots represent the percentage of expected individuals carrying the genotype with one risk allele; red dots represent the percentage of expected individuals carrying the genotype with both risk alleles.

*Means significantly ($p<0.05$) differences of allele distribution of each country compared with Finland (as reference country). Statistical differences have been assessed by Chi-Square.
